# Characterization of the emerging recombinant infectious bronchitis virus in China

**DOI:** 10.3389/fmicb.2024.1456415

**Published:** 2024-10-15

**Authors:** Suchun Wang, Junhui Pan, Kaiyutai Zhou, Dianfeng Chu, Jinji Li, Yiping Chen, Qian Qi, Shimeng Wei, Chao Li, Jinyu Sui, Faxing Wu, Jinping Li, Guangyu Hou, Hualei Liu, Kaicheng Wang

**Affiliations:** ^1^China Animal Health and Epidemiology Center, Qingdao, China; ^2^Key Laboratory of Animal Biosafety Risk Prevention and Control (South), Ministry of Agriculture and Rural Affairs, Qingdao, China; ^3^Key Laboratory of Animal Biosafety, Qingdao, China; ^4^Qingdao Yebio Biological Engineering Co., Ltd., Qingdao, China

**Keywords:** emerging recombinant IBV, epidemiology, characterization, recombination, pathogenicity

## Abstract

Infectious bronchitis virus (IBV) can cause serious harm to poultry industry. It is belong to Coronaviridae which is highly variable. A kind of emerging recombinant IBV (ahysx-1) has been detected in chicken from China in 2016. To understand the epidemiology and characterization of the emerging recombinant IBV, 35,455 samples of chickens from the 15 provinces in China were collected and detected. One hundred and ninety-six out of the 537 flocks (positive rate, 36.49%), and 908 out of 35,455 samples (positive rate, 2.56%) were positive in the detection. The results showed that the emerging recombinant IBV was pandemic in China. Thirteen emerging recombinant IBV isolates were selected and continuous subcultured to the fourth generation and analyzed by Next-generation sequencing. Compared with the reported sequence of ahysx-1, the genomic analysis showed that multiple position insertions and deletions were in 1a gene, 3b gene, M gene and N gene. The identity of the *S* gene nucleotide sequence between all the 13 emerging recombinant IBV isolates and reference stain ahysx-1 were 98.1–99.1%, while the identity of amino acid sequence were 98.0–99.8%. To better understand the recombination mechanism of the emerging recombinant IBV, the genomic sequence of the 13 isolates were compared with turkey coronavirus or guinea fowl coronavirus. The results suggest that all the 13 emerging recombinant IBV isolates were likely to be the recombination of turkey coronavirus or guinea fowl coronavirus with IBV. Turkey coronavirus or guinea fowl coronavirus as minor parents are the donors of S gene. The major parents donors of the genome backone of these recombination events were lineages GI-19 or GVI-1 of IBV. One isolate (IBV/chicken/Henan/H1173/2021) was selected for pathogenicity analysis. The results showed that IBV/chicken/Henan/H1173/2021 was avirulent to SPF embryonated eggs, but could cause intestinal symptoms in of chicks. This study provides a foundation for understanding the epidemic situation and characterization of the emerging recombinant IBV. It is of great significance for the prevention and control of avian coronavirus infection.

## 1 Introduction

Infectious bronchitis (IB) is a highly contagious disease of poultry, which can cause high economic losses in poultry industry (Jackwood, 2012). IBV was first detected in North Dakota, USA, in the 1930s (Schalk and Hawn, 1931). In chickens, IBV damages not only the respiratory system but also the bursae of Fabricius, digestive tract tissues, and the urogenital system. It can cause poor egg quality and even death (Johnson and Marquardt, 1975; Jackwood, 2012).

IBV is a member of the Gammacoronavirus genus, the Coronaviridae family, and the Nidovirales order. The IBV genome consists of a single-stranded positive-senese RNA molecule of approximately 27.6 kb, which encodes four structural proteins, including the spike glycoprotein (S) and envelope (E), membrane (M), and nucleocapsid (N) proteins, as well as four non-structural accessory proteins (3a, 3b, 5a and 5b) (Johnson and Marquardt, 1975). Genetic diversity of IBV is caused by genetic mutations and recombination. The lack of proofreading capabilities of viral RNA-dependent RNA polymerase (RdRp) is responsible for the genetic mutations such as point mutations, insertions and deletions during viral replication (Shi et al., 2000; Liu et al., 2009). Therefore, new genotypes and serotypes of IBV are continually emerging worldwide, even among vaccinated flocks. Genetic analysis based on the S1 gene of IBV has become a common method for virus genotype classification. Until now, phylogenetic classification of IBV strains results in seven genotypes (GI-GVIII) consisting of 37 genetic lineages (Valastro et al., 2016; Jiang et al., 2017; Ma et al., 2019; Molenaar et al., 2020). Genotype GI has the largest number of genetic lineages (n: 29). Genotype GII has two genetic lineages. Genetic recombination, deletion, and insertion drive the evolution of IBV and new variants continue to emerge (Xiong et al., 2024). Several kinds of IBV variants are continuously discovered in recent years (Li et al., 2017; Jang et al., 2018; Domanska-Blicharz et al., 2020). Some recombination events were identified to be between field and vaccine strains (Ren et al., 2020; Gong et al., 2022).

In China, IBV was reported for the first time in the 1980s (Lian et al., 2021). At present, IBV is widespread in poultry. GI-19 (QX), GI-13 (4/91), GVI-1 (TC07-2), GI-7 (TW-I), GI-1 (Mass), and GI-28 (LDT3-A) genotypes are dominant in China (Ji et al., 2020; Lian et al., 2021; Yan et al., 2021). Variant IBVs emerged and have been isolated continuously in China (Xue et al., 2012; Jiang et al., 2020). In 2020, a kind of the emerging recombinant IBV (ahysx-1) with a S gene closely related to that of a turkey coronavirus was detected in a chicken using viral metagenomics (Wang et al., 2020). The S gene of the emerging recombinant IBV showed low similarity (58% identity) to the other IBVs, however higher similarity (>75% identity) to turkey coronaviruses. The skeleton sequences were highly similar (98% identity) to IBV isolates from chickens.

So far, only one genome sequence of the emerging recombinant IBV has been published. The prevalence and characteristics of the virus was unknown. To further investigate the epidemiology and characterization of the emerging recombinant IBV in China, the epidemiology and characterization of the emerging recombinant IBV was analyzed in this study.

## 2 Materials and methods

### 2.1 Ethics statement

This study was conducted according to the animal welfare guidelines of the World Organization for Animal Health (Edgar, 2004), and approved by the Animal Welfare Committee of Qingdao Yebio Biological Engineering Co., Ltd. The approval number was YBFL2022001. Swab and drinking-water samples were all collected with permission given by multiple relevant parties, including the Ministry of Agriculture of China, the China Animal Health and Epidemiology Center, and the relevant veterinary sections in the provincial and county government.

### 2.2 Sample collection and RNA extraction

From 2021 to 2023, a total of 35,455 samples from 20,859 chickens, 9,592 ducks, 2,818 pigeons, 1,973 goose and 193 other birds were collected from the 15 provinces in China, including 35,285 swab samples and 170 drinking-water samples. These samples were collected from 537 live poultry markets (LPMs), poultry farms or slaughter houses. The swab samples were smears taken from both the cloacal and oropharyngeal tracts of poultry (Wang et al., 2017). All the 35,455 samples were clarified by centrifugation at 10,000 rpm for 5 min, and then the supernatants were inoculated in 10-day-old specific-pathogen-free (SPF) chicken embryonated eggs (Shandong Healthtec Laboratory Animal Breeding Company, Jinan, China) via the allantoic sac route. The inoculated eggs were incubated at 37°C for 3 days. After the incubation period, the allantoic fluids of all the inoculated eggs were collected and centrifuged at 12,000 rpm, 4°C for 10 min. The genomic RNA was extracted from 200 μL supernatants of each sample with the QIAxtractor platform using a cador Pathogen 96 QIAcube HT kit (Qiagen, Hilden, Germany) according to manufacturer’s instructions. All the extracted RNA samples were stored at −80°C.

### 2.3 Emerging recombinant IBV detection

To detect and analyze the emerging recombinant IBVs with the genomic characterization that the S gene is higher homology with Turkey coronavirus, while other genes is higher homology with IBVs, a pair of primers was developed to amplify a 1,373-base fragment including partial S gene (1,187 bases). The forward primer was designed on the 1b gene, and the reverse primer was designed on the s1 gene of ahysx-1 (GenBank number: MK142676). The RT-PCR method could only detect the emerging recombinant strains but not the non-recombinant strains. The forward primer was rIBV-F: 5′-AAAACM(A/C)CTGCACGCAAATTA-3′, and the reverse primer was rIBV-R 5′-TCAAAACCTTCR(A/G)ACACTAACAATA-3′. RT-PCR was performed by Evo M-MLV One Step RT-PCR Kit (Accurate Biotechnology (Hunan) Co., Ltd., Hunan, China; AG11606). The reaction system included 12.5 μL of 2× One-step Master Mix, 1 μL each of the upstream and downstream primers (10 pM), 1 μL of Evo M-MLV RTase, 3 μL of nucleic acids, and 6.5 μL of RNase Free dH_2_O. RT-PCR was performed with incubation at 50°C for 30 min, followed by denaturation at 94°C for 2 min, and 30 cycles of 94°C for 30 s, 55°C for 30 s, 72°C for 2 min, and 72°C for 5 min. The RT-PCR products were purified with an agarose gel DNA extraction kit, and sequenced directly with the ABI 3730xl DNA Analyzer (Sangon, Shanghai, China). All the extracted RNA from the 35,455 samples were detected. The specific RT-PCR products were identified as emerging recombinant IBV by sequencing and comparison with the reference sequence of ahysx-1.

### 2.4 Subculture of the emerging recombinant IBVs

A total of 106 positive samples collected from different regions were selected for continuous subculture, and their pathogenicity to SPF chicken embryo was observed at the same time. Only 13 emerging recombinant IBV isolates could be continuous subcultured to the fourth generation and negative in the Avian influenza virus (AIV), Newcastle disease virus (NDV) and J subgroup avian leukemia virus (ALV-J) detection. AIV, NDV and ALV-J were detected by RT-qPCR using the *Evo M-MLV* One Step RT-qPCR Kit II (Accurate Biotechnology (Hunan) Co., Ltd., Changsha, China; AG11713). The primers and probes in each test reaction are shown in Supplementary Table S1. The reaction system included 10 μL of 2× One Step RT-qPCR Buffer II (Probe), 0.4 μL each of the upstream and downstream primers (10 pM), 0.4 μL of probe (10 pM), 0.4 μL of *Evo M-MLV* RTase Enzyme Mix II, 0.4 μL of *Pro Taq* HS DNA Polymerase, 2 μL of nucleic acids, and 6 μL of RNase Free dH_2_O. The reaction conditions were as follows: reverse transcription for 5 min at 42°C, followed by 30 s at 94°C, followed by 40 cycles of denaturation for 5 s at 94°C, annealing and extension for 30 s at 60°C. Nucleic acids of the related viruses were also detected as positive controls. The detection results were determined according to the judgment standards in published paper listed in Supplementary Table S1. The fourth generation allantoic fluid of the 13 isolates were collected and analyzed in NGS and animal experiments.

### 2.5 Genome sequencing of the emerging recombinant IBV

The sequencing libraries of the selected 13 emerging recombinant IBV isolates genome were constructed using the Ion Total RNA-Seq kit v2 (Qiu et al., 2019). The DNA library was pretreated using the Ion Personal Genome Machine (PGM) Template OT2 200 Kit (Life Technologies, USA). The libraries were loaded onto an Ion 318 chip and sequenced using the Ion Torrent PGM platform (Life Technologies) with the Ion PGM Sequencing 200 Kit (Life Technologies, USA) (Merriman and Rothberg, 2012). Quality control was autorun by the Ion Torrent PGM platform to delete some low quality reads. The sequencing adaptor of reads was automatically deleted using Torrent Server. The Ion Torrent PGM singleton reads of each library were assembled according to the reference sequence of ahysx-1 (GenBank accession number: MK142676) by CLC genomics workbench (version 12).

### 2.6 Phylogenetic analysis of the emerging recombinant IBV genome

The assembled emerging recombinant IBV sequences obtained in this study were analyzed using MEGA 6.0. The genome sequences and S gene, M gene, N gene of the 13 emerging recombinant IBV isolates were aligned with 84 genome reference sequences (list in Supplementary Table S2) of Alphacoronavirus, Betacoronavirus, Gammacoronavirus and Deltacoronavirus available in GenBank using the software MUSCLE (Edgar, 2004). Bayesian information criterion (BIC) scores of substitution models and phylogenetic relationships were calculated using the software package MEGA 6.0 (Hall, 2013; Tamura et al., 2013). Phylogenetic relationships were calculated using the model with the lowest BIC score which is assumed to describe the substitution pattern the best. Gaps were handled by partial deletion and bootstrap values were calculated out of 1,000 replicates (Hall, 2013).

### 2.7 Characterization analysis of *S* gene, *M* gene and *N* gene

Antigenic epitopes in the S1 and S2 subunits of S protein of the emerging recombinant IBVs were predicted and analyzed by the online prediction tool.^1^ It has been reported that the method has an accuracy of about 75%, which could predict the sequence of possible antigen fragments by simulating the antibody response (Kolaskar and Tongaonkar, 1990).

The M protein transmembrane region of the emerging recombinant IBVs were predicted and analyzed by transmembrane hidden Markov model (TMHMM) using the DTU Bioinformatics online prediction tool.^2^ TMHMM is one of the most complete and best-performing topological structure prediction methods for transmembrane proteins at present (Chen et al., 2003; Nugent and Jones, 2009). In this study, the original amino acid sequence is encoded through the pre-trained model, and then the topological structure is decoded through the prediction space model. The transmembrane region can be predicted with high accuracy. Seventeen kinases (ATM, CKI, CKII, CaM-II, DNAPK, EGFR, GSK3, INSR, PKA, PKB, PKC, PKG, RSK, SRC, cdc2, cdk5 和 p38MAPK) were predicted and the phosphorylation site of N protein in the emerging recombinant IBV isolates were analyzed by the online tool^3^ (Dinkel et al., 2011).

### 2.8 Recombination analysis of the emerging recombinant IBV genome

The recombination of the emerging recombinant IBVs were analyzed by Recombination Detection Programme 4 (RDP4) with various genotypes of IBVs, turkey coronaviruses and guinea fowl coronaviruses, respectively, (Martin et al., 2015). In order to ensure the accuracy of composite analysis, seven calculation methods are used. The calculation result was judged to reliable when *p*-value ≤10^−12^ (*p* < 0.05). When at least five of the seven calculation results were credible, recombination event was considered to be occurred (Thor et al., 2011).

### 2.9 Pathogenicity assays in SPF chickens

To understand the pathogenicity of the emerging recombinant IBVs, one isolate (IBV/chicken/Henan/H1173/2021) was selected for SPF chickens infection experiments. SPF 1-day-old White Leghorn chickens provided by Qingdao Yebio Biological Engineering Co., Ltd. were randomly divided into two groups of 10 chickens each. One group of SPF chickens was infected with the selected emerging recombinant IBV isolate via intranasally and eye drop inoculation route using 200 μL inoculum (10^2.65^ EID_50_/0.1 mL) (Callison et al., 2006). The other group was inoculated with sterile PBS as negative controls. All chickens were observed twice a day for clinical symptom including tracheal rales, nasal discharge, coughing, eye irritation and watery diarrhea. Morbidity and mortality were recorded every day.

## 3 Results

### 3.1 Epidemiology of emerging recombinant IBV

A total of 908 sequences were obtained and align with the reference sequence of ahysx-1. The similarity between 908 sequences to ahysx-1 stain ranged from 94.1 to 100%. Therefore, the corresponding 908 samples were considered positive for the emerging recombinant IBV. One hundred and ninety-five out of the 537 flocks (group positive rate, 36.49%) and 908 out of the 35,455 samples (individual positive rate, 2.56%) tested positive for emerging recombinant IBV. The 908 emerging recombinant IBV positive samples were obtained from 869 chickens, 22 ducks, 14 pigeons and three geese. The emerging recombinant IBVs have been detected in 14 out of 15 provinces. The detection results were listed in Table 1. The emerging recombinant IBV was widely distributed in flocks, suggesting that the virus was prevalent in China. Chickens are the main host of the emerging recombinant IBV.

**Table 1:** Detection results of 15 provinces for the emerging recombinant IBV.

Province	Chickens^a^	Ducks	Pigeons	Geese	Total
Anhui	112/1,407 (7.96%)	3/1,006 (0.30%)	1/403 (0.25%)	0	116/2,880 (4.03%)
Fujian	45/1,264 (3.56%)	2/1,368 (0.15%)	1/213 (0.47%)	0	48/2,870 (1.67%)
Guangdong	61/1,226 (4.98%)	1/937 (0.11%)	0	0	62/3,180 (1.95%)
Guangxi	46/1,103 (4.17%)	3/1,271 (0.24%)	0	0	49/2,880 (1.70%)
Guizhou	33/519 (6.36%)	0			33/960 (3.44%)
Henan	47/2,365 (1.99%)	2/217 (0.92%)	0	0	49/2,872 (1.71%)
Heilongjiang	3/540 (0.56%)	0	0	0	3/953 (0.31%)
Hubei	104/1,935 (5.37%)	0	1/137 (0.73%)	0	105/3,212 (3.27%)
Hunan	53/1,060 (5.00%)	1/734 (0.14%)	0	0	54/1,918 (2.82%)
Jiangsu	76/2,166 (3.51%)		0	0	76/2,880 (2.64%)
Jiangxi	111/1,383 (8.03%)	6/1,153 (0.52%)	1/467 (0.21%)	3/27 (11.11%)	121/3,030 (3.99%)
Ningxia	16/1,537 (1.04%)	0	0	0	16/1,880 (0.85%)
Shandong	0	0	0	0	0
Sichuan	86/1,585 (5.43%)	1/476 (0.21%)	0	0	87/2,400 (3.63%)
Yunnan	76/2,304 (3.30%)	3/347 (0.86%)	10/113 (8.85%)	0	89/2,820 (3.16%)
Total	869/20,859 (4.17%)	22/9,592 (0.23%)	14/2,818 (0.50%)	3/1,973 (0.15%)	908/35,455 (2.56%)

^a^Number of positive samples/total number of samples (positive rate).

### 3.2 Genome characterization and phylogenetic analysis

The 13 emerging recombinant IBV isolates were sequenced by next-generation sequencing. The genomes were all sequenced by PGM in a single run with the sequencing depth more than 500. The genomic information of the 13 isolates, such as sequence length and GenBank accession numbers were listed in Table 2.

**Table 2:** Genome information of 13 emerging recombinant IBVs isolated in this study.

Strain	Complete genome fragment length (bp)	GenBank accession number	Percent similarity of the major structural genes between the 13 emerging recombinant IBVs and ahysx-1 (%)					
			S gene (nucleotide)	S gene (amino acid)	M gene (nucleotide)	M gene (amino acid)	N gene (nucleotide)	N gene (amino acid)
IBV/chicken/Anhui/A1214/2021	27,788	OP881969	98.7	99.1	97.5	97.8	97.4	98.0
IBV/chicken/Sichuan/C1131/2021	27,732	OP881970	98.5	98.4	99.1	99.1	98.0	98.0
IBV/chicken/Jiangxi/E1197/2021	27,779	OP881971	98.7	99.1	99.3	99.6	97.6	98.5
IBV/chicken/Jiangxi/E1471/2021	27,786	OP881972	99.1	99.5	99.1	99.6	95.2	96.8
IBV/chicken/Gungdong/G1225/2021	27,749	OP881973	98.9	99.3	99.1	99.1	97.0	97.1
IBV/chicken/Henan/H1173/2021	27,786	OP881974	99.1	99.8	99.6	99.6	96.8	97.6
IBV/chicken/Ningxia/N1379/2021	27,736	OP881975	98.1	98.0	93.5	95.5	95.4	95.9
IBV/chicken/Yunnan/Y1389/2021	27,731	OP881976	98.6	99.1	99.4	99.6	96.2	97.3
IBV/chicken/Jiangxi/E1068/2021	27,780	OP881977	98.8	99.5	99.1	99.6	96.2	96.8
IBV/chicken/Jiangxi/E1205/2021	27,781	OP881978	98.5	99.3	99.4	99.6	96.7	97.3
IBV/chicken/Jiangxi/E1283/2021	27,757	OP881979	99.1	99.6	99.3	99.6	96.3	97.6
IBV/chicken/Jiangxi/E1201/2021	27,786	OP881980	98.8	99.5	99.1	99.6	95.8	96.8
IBV/chicken/Jiangxi/E1207/2021	27,786	OP881981	98.5	99.3	99.4	99.6	95.9	96.6

**Figure 1:**
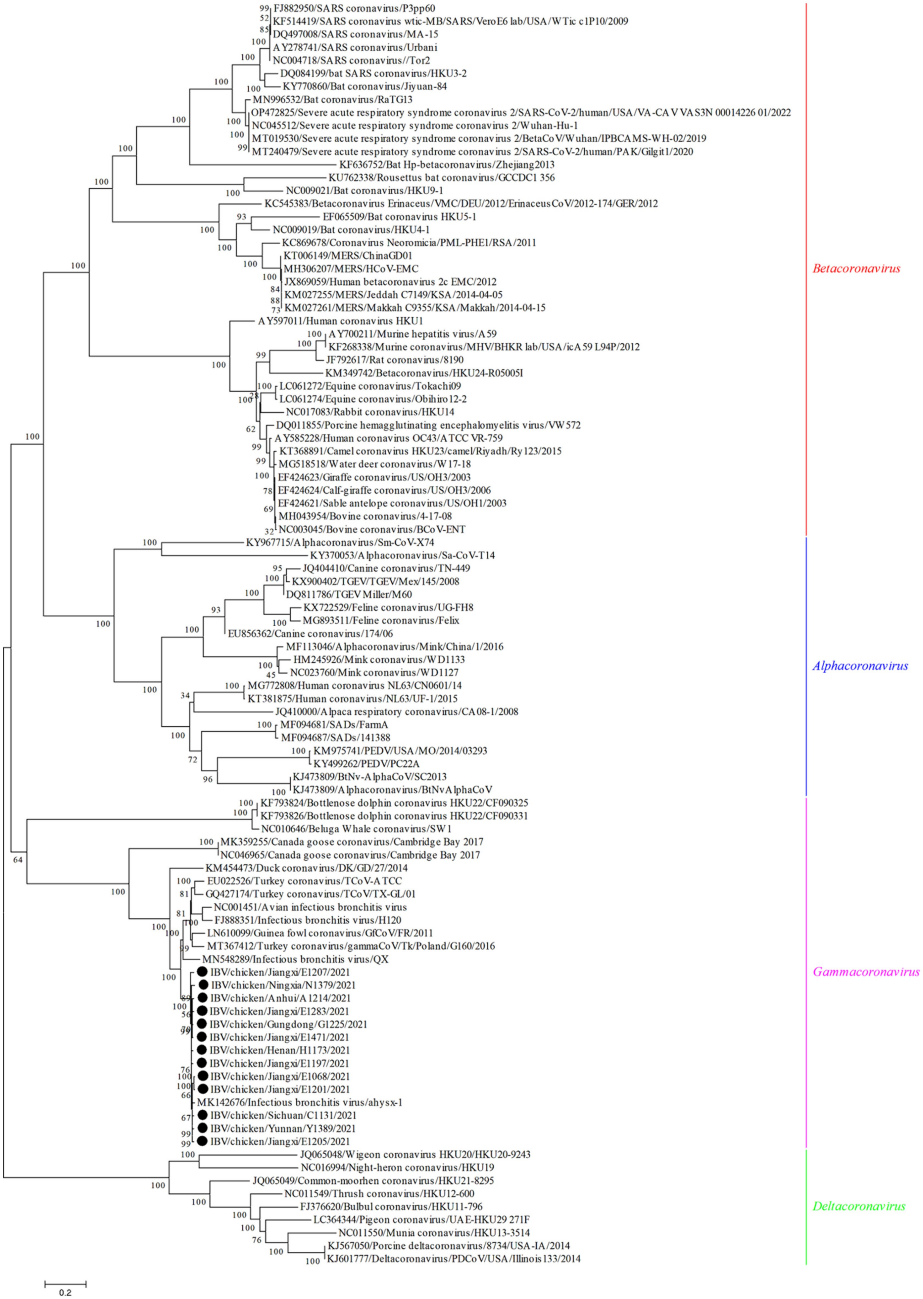
Phylogenetic analysis of genome sequences of 13 emerging recombinant IBVs. The sequences were aligned using Clustal W. The trees were constructed using the model with the maximum likelihood (ML) method, gaps were handled by partial deletion and bootstrap values were calculated out of 1,000 replicates. The 13 emerging recombinant IBV isolates detected in this study were labeled by black solid circles “●.” All the 13 emerging recombinant IBVs belong to *Gammacoronavirus.*

**Figure 2:**
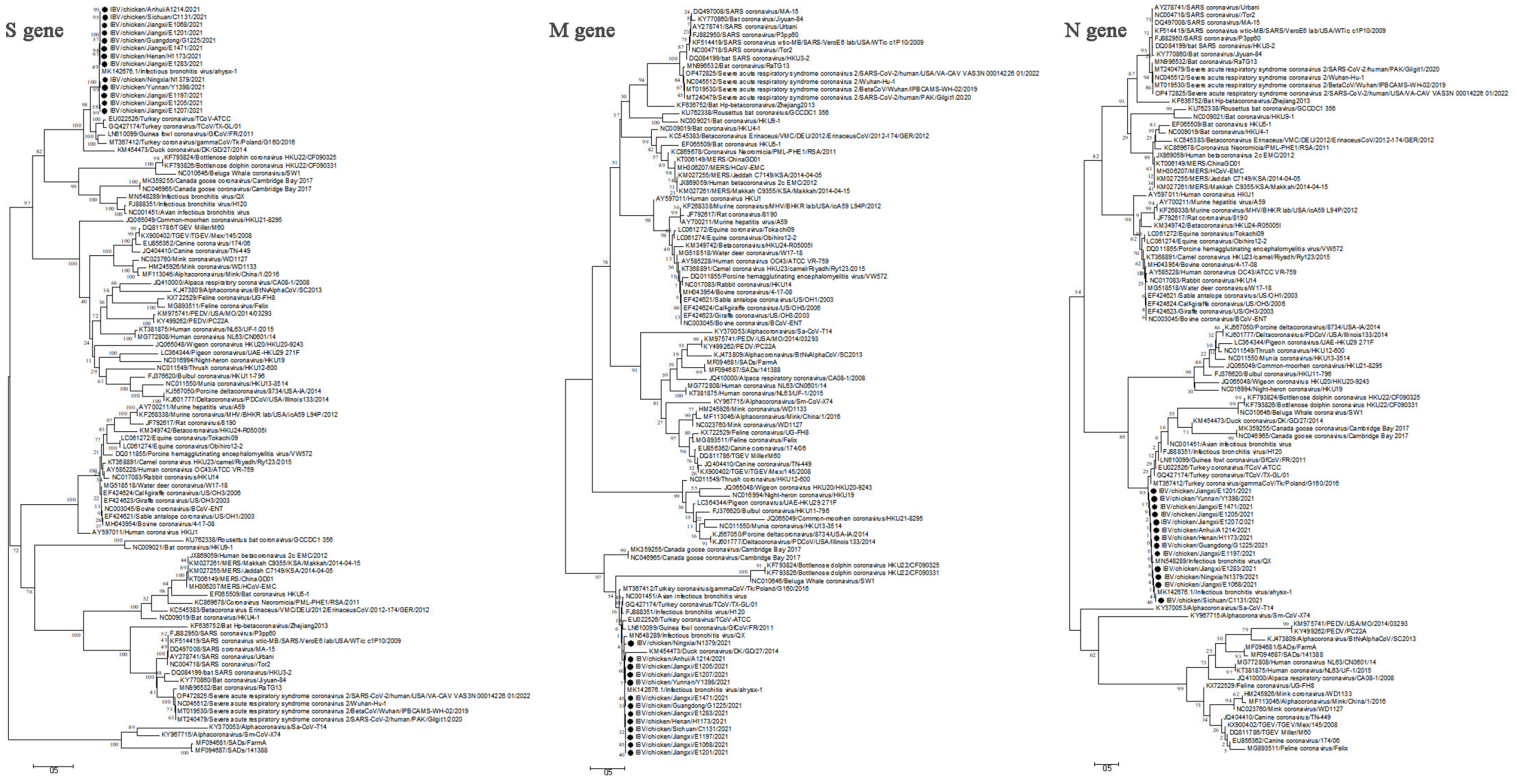
Phylogenetic analysis of *S* gene, *M* gene and *N* gene of 13 emerging recombinant IBVs. The sequences were aligned using Clustal W. The trees were constructed using the model with the maximum likelihood (ML) method, gaps were handled by partial deletion and bootstrap values were calculated out of 1,000 replicates. The 13 emerging recombinant IBV isolates detected in this study were labeled by black solid circles “●.” The *S* gene, *M* gene and *N* gene of 13 emerging recombinant IBVs were all derived from *Gammacoronavirus.*

The genome sequences analysis were performed to further investigate the characterization and phylogenetic of the emerging recombinant IBVs. Sequence analysis results demonstrated that the genome structure of 13 emerging recombinant IBVs was 5′-1a,b-S(S1,S2)-3a,b,c(E)-M-5a,b-N-Poly(A)-3′, which was consistent with the reported isolate ahysx-1. Some insertions and deletions were discovered during the analysis of nucleotide sequences with ahysx-1 strain and common used IBV reference stains (Supplementary Figure S1). IBV/chicken/Jiangxi/E1283/2021 and IBV/chicken/Gungdong/G1225/2021 have deletions of 27 nucleotides and 30 nucleotides in the 1a gene which similar to H120 strain (GenBank accession number: NC001451), respectively. Three isolates have deletions of 27 nucleotides in the 3a and 3b genes which similar to ahysx-1strain but different from the common IBV reference strains. The 13 emerging recombinant IBV isolates have two specific deletions of 9 and 3 nucleotides in M gene respectively, similar to ahysx-1 strain but different from the common IBV reference strains. IBV/chicken/Gungdong/G1225/2021 have specific deletion of three nucleotides in the *N* gene different from other 12 emerging recombinant IBV isolates, ahysx-1 strain and the common IBV reference strains. The percentage of nucleotide sequence similarity of the 13 emerging recombinant IBV isolates is 97.61% on average. Phylogenetic analysis results showed that all the 13 emerging recombinant IBVs belong to *Gammacoronavirus* (Figure 1). The *S* gene, *M* gene and *N* gene of 13 emerging recombinant IBVs were all derived from *Gammacoronavirus* (Figure 2).

### 3.3 Characterization and phylogenetic analysis of the *S* gene, *M* gene, *N* gene

The gene sequences of the major structural proteins of the 13 emerging recombinant IBV isolates were highly homologous. In this study, all the *S* gene of 13 isolates contained 3,618 nucleotides which could encode 1,206 amino acids. All the *M* gene of 13 isolates contained 672 nucleotides which could encode 224 amino acids. Furthermore, all the *N* gene of 13 isolates contained 1,230 nucleotides which could encode 410 amino acids The percent similarity of *S* gene, *M* gene, *N* gene between the 13 emerging recombinant IBVs and ahysx-1 were listed in Table 2.

The cleavage site of S protein in the 13 emerging recombinant IBV isolates was located at the same amino acid position 537–542. The nucleotide sequence of S1 gene is being widely used to analyze the genetic evolution of IBV. The results showed that the number of peptide in the S1 subunit of 13 isolates was different, while in the S2 subunit was identical. The average propensities of S protein, S1 subunit and S2 subunit were all greater than 1.0, indicating that all of them had potentially antigenic (Supplementary Table S3). It was speculated that the 13 isolates have one potential site for amino acid glycosylation at the same amino acid position. Three transmembrane regions were predicted at the N-terminal of M protein of 13 isolates (Supplementary Figure S2 and Supplementary Table S4). The structural characteristics of M protein were consistent with 13 isolates. Phosphorylated modification was found to be require for the processing of N protein. The phosphorylation sites of N protein in the 13 isolates ranged from 41 to 44.

### 3.4 Recombination analysis of the 13 emerging recombinant IBV isolates

The recombination of the 13 emerging recombinant IBVs were analyzed by RDP4 with a total of 68 various genotypes of IBVs, turkey coronaviruses and guinea fowl coronaviruses from GenBank, respectively. The results showed that the *S* gene of the 13 isolates may have undergone recombination events. It suggested that turkey coronavirus (GenBank accession number: EU022526, GQ427174, GQ427176) and guinea fowl coronavirus (GenBank accession number: LN610099) might be the main donors of S gene. The major parents donors of the genome backone of these recombination events were genotype GI-19 or GVI-1 of IBV (Supplementary Figure S3 and Table 3). The results of recombination analysis were similar to the previously reported finding (Wang et al., 2020).

**Table 3:** The *p* values of different methods in each recombination event.

Recombinant sequence	Minor parental sequence	Major parental sequence	Recombinant gene	The *p* values of seven methods in each recombination event						
				RDP	GENECONV	Bootscan	Maxchi	Chimaera	SiSscan	3Seq
IBV/chicken/Anhui/A1214/2021	GQ427174 TCoV/TXGL/01	KX259254 ckCH-LSD-090314	S	2.84 × 10^−76^	3.83 × 10^−135^	8.68 × 10^−71^	4.33 × 10^−67^	1.18 × 10^−43^	1.01 × 10^−78^	2.80 × 10^−14^
IBV/chicken/Sichuan/C1131/2021	LN610099 GfCoV/FR/2011	KX348115 ck/CH/LAH/120907	S	1.41 × 10^−101^	3.43 × 10^−165^	3.04 × 10–^171^	1.02 × 10^−74^	7.85 × 10^−44^	2.53 × 10^−107^	1.24 × 10^−14^
IBV/chicken/Jiangxi/E1197/2021	EU022526 TCoV-ATCC	KY421672 SZ	S	1.31 × 10^−158^	6.54 × 10^−181^	3.27 × 10^−129^	5.97 × 10^−74^	6.38 × 10^−43^	2.90 × 10^−107^	1.01 × 10^−13^
IBV/chicken/Jiangxi/E1471/2021	GQ427176 TCoV/TX/1038/98	KX348115 ck/CH/LAH/120907	S	1.83 × 10^−115^	5.32 × 10^−157^	6.67 × 10^−136^	2.83 × 10^−76^	3.08 × 10^−43^	1.33 × 10^−96^	4.88 × 10^−14^
IBV/chicken/Gungdong/G1225/2021	GQ427176 TCoV/TX/1038/98	KX640829 ck/CH/LGX/111119	S	5.31 × 10^−158^	2.19 × 10^−193^	3.75 × 10^−183^	5.74 × 10^−80^	4.90 × 10^−44^	3.87 × 10^−119^	7.77 × 10^−15^
IBV/chicken/Henan/H1173/2021	EU022526 TCoV-ATCC	KY421672 SZ	S	4.87 × 10^−163^	7.18 × 10^−198^	2.74 × 10^−133^	3.04 × 10^−09^	4.01 × 10–^43^	3.02 × 10^−107^	6.35 × 10^−14^
IBV/chicken/Ningxia/N1379/2021	LN610099 GfCoV/FR/2011	KX640829 ck/CH/LGX/111119	S	7.74 × 10^−100^	5.30 × 10^−173^	9.42 × 10^−110^	1.88 × 10^−75^	2.31 × 10^−43^	4.95 × 10^−98^	3.66 × 10^−14^
IBV/chicken/Yunnan/Y1389/2021	GQ427176 TCoV/TX/1038/98	MW791835 CK/CH/FJ/202005	S	1.57 × 10^−169^	4.07 × 10^−189^	8.36 × 10^−204^	5.09 × 10^−76^	2.31 × 10^−43^	4.63 × 10^−121^	3.66 × 10^−14^
IBV/chicken/Jiangxi/E1068/2021	EU022526 TCoV-ATCC	KX259254 ck-CH-LSD-090314	S	1.38 × 10^−156^	1.48 × 10^−161^	5.94 × 10^−117^	1.14 × 10^−76^	1.68 × 10^−43^	6.80 × 10^−98^	2.66 × 10^−14^
IBV/chicken/Jiangxi/E1205/2021	EU022526 TCoV-ATCC	KX236004 ck/CH/LHLJ/150701	S	1.87 × 10^−157^	8.67 × 10^−163^	3.06 × 10^−185^	5.91 × 10^−68^	7.85 × 10^−43^	5.61 × 10^−99^	1.87 × 10^−13^
IBV/chicken/Jiangxi/E1283/2021	GQ427174 TCoV/TXGL/01	KX252787 ck/CH/LLN/131040	S	1.60 × 10^−85^	3.62 × 10^−137^	7.63 × 10^−106^	4.75 × 10^−69^	3.08 × 10^−43^	4.79 × 10^−93^	4.88 × 10^−14^
IBV/chicken/Jiangxi/E1201/2021	EU022526 TCoV-ATCC	KX348115 ck/CH/LAH/120907	S	1.29 × 10^−63^	1.34 × 10^−146^	3.90 × 10^−92^	5.26 × 10^−74^	3.08 × 10^−43^	1.13 × 10^−98^	4.88 × 10^−14^
IBV/chicken/Jiangxi/E1207/2021	GQ427174 TCoV/TXGL/01	KX259254 ck-CH-LSD-090314	S	5.42 × 10^−99^	7.41 × 10^−127^	3.50 × 10^−118^	4.65 × 10^−69^	3.08 × 10^−43^	2.05 × 10^−56^	NS

### 3.5 Pathogenicity analysis of the emerging recombinant IBVs

There were no classical symptoms such as enbryo stunting, curling, urate deposits in the mesonephros of the kidney or death in the continuous subculture of the selected IBV/chicken/Henan/H1173/2021 isolate. The results revealed that the IBV/chicken/Henan/H1173/2021 isolate was avirulent to SPF embryonated eggs. Pathogenicty analysis results in the SPF chickens indicated that all the 10 chickens in the infected group brought about mild diarrhea, but did not die. All chicks appeared paste anus on day 4 (Supplementary Figure S4). At necropsy the intestine revealed congestion but no necrotic foci (Supplementary Figure S4). In control group, all the SPF chickens had no clinical symptoms and no death. All oropharyngeal and cloacal swab samples detected positive with the Ct value ranging from 16.31 to 27.83. In conclusion, IBV/chicken/Henan/H1173/2021 isolate continuous to be excreted through oropharynx or /and cloaca after infected the SPF chickens.

## 4 Discussion

In this study, the epidemiology and characterization of the emerging recombinant IBVs were analyzed for the first time. The results showed that 196 out of the 537 flocks (positive rate, 36.49%), and 908 out of the 35,455 samples (positive rate, 2.56%) were detected to be positive for emerging recombinant IBV. According to the published paper, the positive rate of IBV was 15.35% in the healthy flocks, and 33.1% in the examined flocks showing respiratory symptoms from China (Li et al., 2021; Lian et al., 2021). Although the individual positive rate of the emerging recombinant IBV was significantly lower than that of IBV, the emerging recombinant IBV has been detected in 14 out of 15 provinces, suggesting that the virus was prevalent in poultry flocks in China. Similar to IBVs, the main host of emerging recombinant IBVs is chicken. Therefore, surveillance should be focused on chicken flocks to understand the prevalence and variation of emerging recombinant IBVs.

Thirteen emerging recombinant IBV isolates were selected for sequencing by NGS to analyze the genetic evolution and biological characteristics. Genome phylogenetic analysis showed that all the 13 isolates belonged to *Gammacoronavirus*. Although some insertions and deletions were discovered during the analysis of nucleotide sequences, the 13 isolates with high homology. The data also indicate the S gene of the 13 isolates had higher homology with turkey coronavirus than IBV. Therefore, the recombination analysis of 13 isolates was carried out by RDP4. The results showed that 13 isolates may have occurred recombination events with the turkey coronaviruses (11/13) or guinea fowl coronaviruses (2/13) as the minor parent and IBVs as the major parent. Turkey coronavirus or guinea fowl coronavirus were donors of *S* gene. Interestingly, 10 out of 13 parental IBV strains as the donor of genome backbone belonged to lineage GI-19, which is similar to the previously reported (Wang et al., 2020). The other three IBV strains belonged to lineage GVI-1 which has not been reported previously.

According to the reports, the average substitution rate for coronavirus was about 10^−4^ substitutions per site 1 year which was high compared to other single-stranded RNA viruses (Pyrc et al., 2006). The cross-host transmission of coronavirus may lead to an increased incidence of recombination events (Pyrc et al., 2006; Woo et al., 2006). In addition, the mutations and recombinations of the large RNA genome in coronavirus can increase the probability for the emergence of novel coronavirus under the right conditions (Pyrc et al., 2006; Su et al., 2016). Furthermore, SARS-CoV-2 is fueled by the emergence of more infectious Omicron variants. Ongoing concerns of emergent variants include possible recombination, as genome recombination is an important evolutionary mechanism for the emergence and re-emergence of human viral pathogens. Therefore, monitoring the evolving coronavirus, including IBV and SARS-CoV-2, genomes especially for recombination is critically important for recognition of abrupt changes to viral attributes including its epitopes which may call for vaccine modifications.

High gene mutation rate and recombination are the important factor to the genetic diversity of IBV (Jackwood et al., 2012). Recombination may create greater genetic variation and lead to the emergence of new strains (Worobey and Holmes, 1999). Potential recombination sites were found in the S proteins of IBV strains, and point mutations in the S1 gene were common, which promotes the evolution of IBV. In recent years, recombination of IBV isolates occurred frequently, with some recombination occurred between vaccine and field isolates (Lee and Jackwood, 2000, 2001; Feng et al., 2017; Li et al., 2017; Zhang et al., 2020).

The IBV/chicken/Henan/H1173/2021 isolate was selected for pathogenicity assay. The results showed that this isolate had no virulence to SPF embryonated eggs. However, the IBV/chicken/Henan/H1173/2021 isolate could cause intestinal symptoms which similar to the clinical symptoms of nephrotropic IBV isolates. The above results showed that the phenotype and pathogenicity of the emerging recombinant IBV isolates may be inconsistent. Because IBV often co-infection with other avian respiratory pathogens in poultry, although the emerging recombinant IBV showed no virulence or low virulence to SPF enbryonated eggs, the further verification is needed to determine whether synergistic effects occur when the emerging recombinant IBV co-infection with other pathogens. IBV and H9N2 AIV are frequently identified in chickens with respiratory symptoms (Kong et al., 2021). Studies have shown that co-infection of IBV and AIV-H9N2 is the most prevalent combination in broiler flocks, can lead to severe clinical symptoms and high mortality (Hassan et al., 2017; Abdelaziz et al., 2019). Therefore, the joint surveillance of IBV and AIV H9N2 will propose effective prevention and control of respiratory disease in poultry.

At present, the prevention and control of IBV mainly rely on the vaccine immunization in China (Feng et al., 2015). Live attenuated vaccines produced by the lineages GI-1 (H120, H52, W93, 28/86, Ma5 and LDT3-A strains), GVI-1 (793B strain) and GI-19 (QXL87 strain) are widely used. The emerging recombinant IBV was detected for the first time from healthy poultry feces in China in 2016 (Wang et al., 2020). In this study, the flocks positive rate of emerging recombinant IBV in poultry has reached 36.49% in China; the emerging recombinant IBVs were detected in almost all the 15 provinces (14/15). The genomic characterization of emerging recombinant IBV is very different from the IBV vaccines used in China. Whether the existing IBV vaccines can produce immune protection against the emerging recombinant IBV was unknown. The pathogenic mechanism of the emerging recombinant IBV should be researched in the further. Further research is needed to develop the new vaccines and antiviral agents should for the prevention and control of the emerging recombinant IBV.

## 5 Conclusion

In this study, the epidemiological surveillance of emerging recombinant IBV were conducted, and the results indicated that the virus was pandemic in China. The genome sequences of 13 emerging recombinant IBV isolates were obtained by Next-generation sequencing. The analysis results of genome sequence showed that the *S* gene of 13 emerging recombinant IBV isolates were highly homologous to the reported ahysx-1 isolate. These 13 emerging recombinant IBV isolates may be formed by recombination of turkey coronaviruses or guinea fowl coronaviruses with IBVs, and could lead to intestinal symptoms in chicks. The spread of the emerging recombinant IBV may increase the difficulty of prevention and control of IB. This study provides a foundation for further research the epidemiology, genetic variation, vaccination and molecular evolutionary relationships of IBV.

## Data Availability

All relevant data are within the manuscript and Supplementary material.

## Footnotes

^1^http://imed.med.ucm.es/Tools/antigenic.pl

^2^https://services.healthtech.dtu.dk/service.php?TMHMM-2.0

^3^https://services.healthtech.dtu.dk/service.php?

## Supplementary material

Information on primers and probes of RT-qPCR assay for AIV, NDV and ALV-J were shown in Supplementary Table S1. Genome information of the 13 recombinant IBVs isolated in this study were shown in Supplementary Table S2. Antigenic epitope prediction analysis of S protein of 13 emerging recombinant IBVs were shown in Supplementary Table S3. The M protein transmembrane region prediction of 13 emerging recombinant IBVs were shown in Supplementary Table S4 and Supplementary Figure S2. The bootscan analysis of 13 recombination events were shown in Supplementary Figure S3. The insertions and deletions of the 13 emerging recombinant IBV strains with reference sequences were shown in Supplementary Figure S1. The clinical symptoms of chicks after inoculation were shown in Supplementary Figure S4.
