# Supplementary figures and images for: Characterization of the emerging recombinant infectious bronchitis virus in China
Source: Front Microbiol. 2024 Oct 15;15:1456415. doi: 10.3389/fmicb.2024.1456415 (PMC11518803; doi:10.3389/fmicb.2024.1456415)

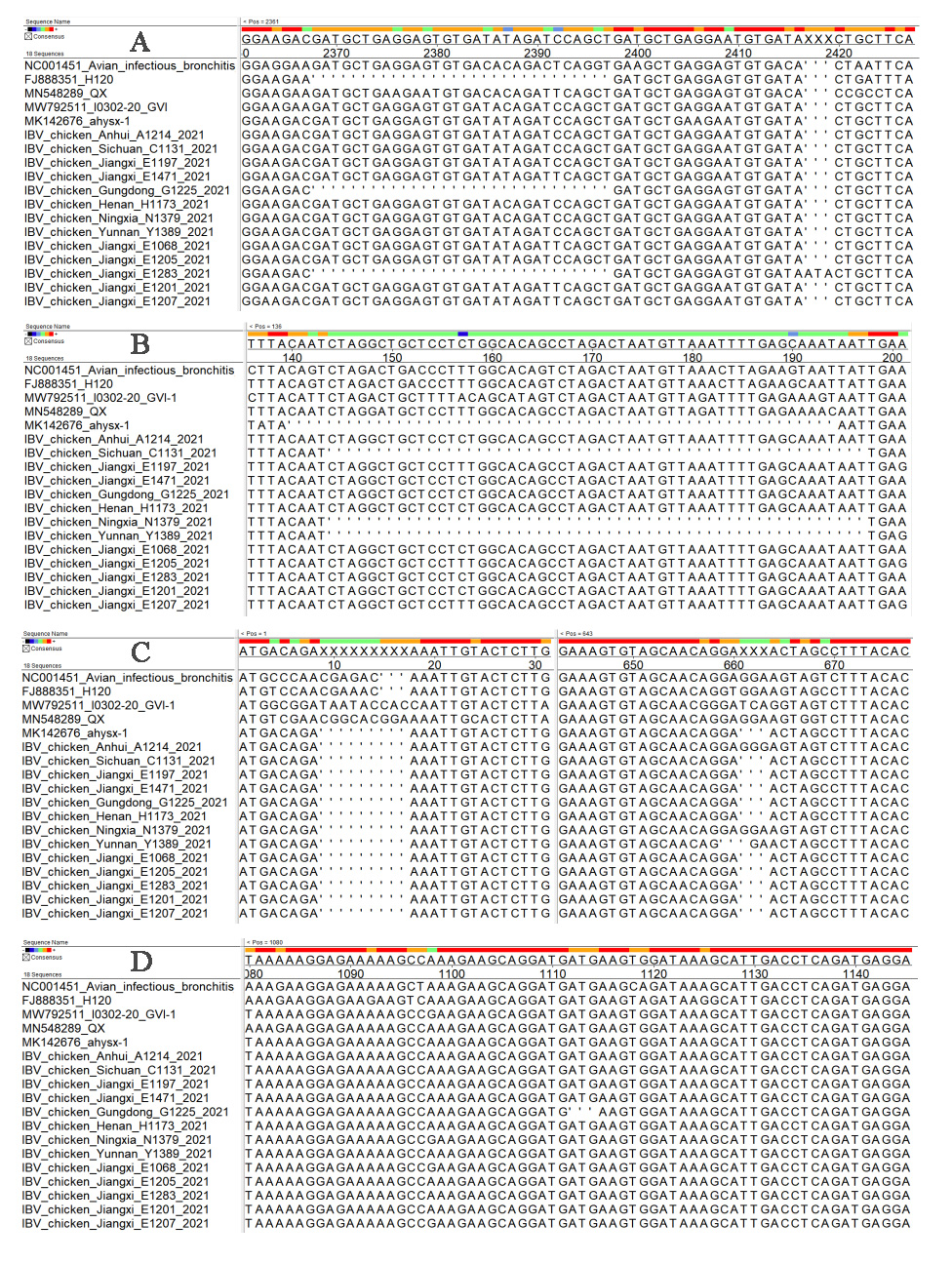

Supplement: Supplementary file 1 [file Image_1.JPEG]

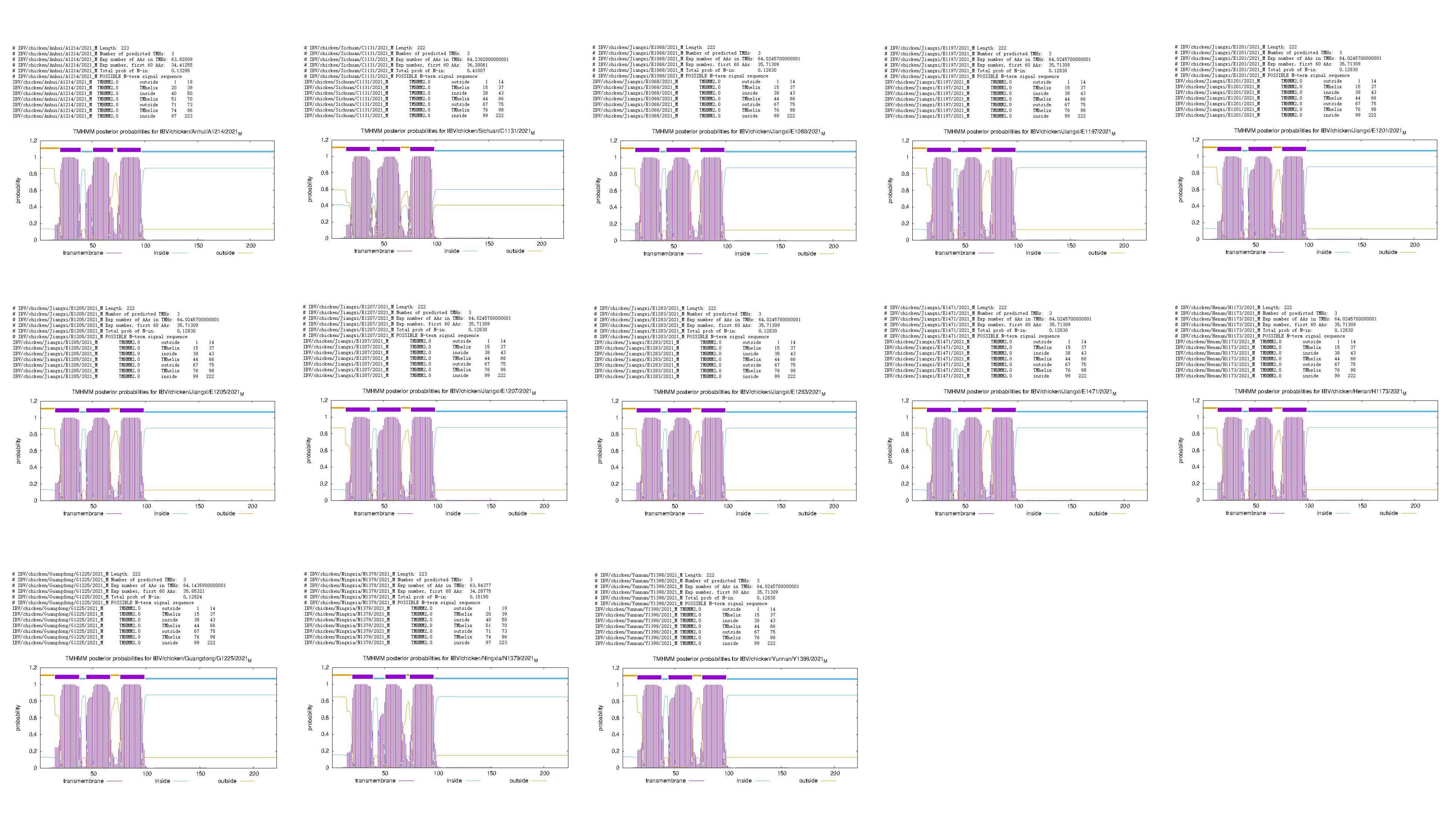

Supplement: Supplementary file 2 [file Image_2.jpg]

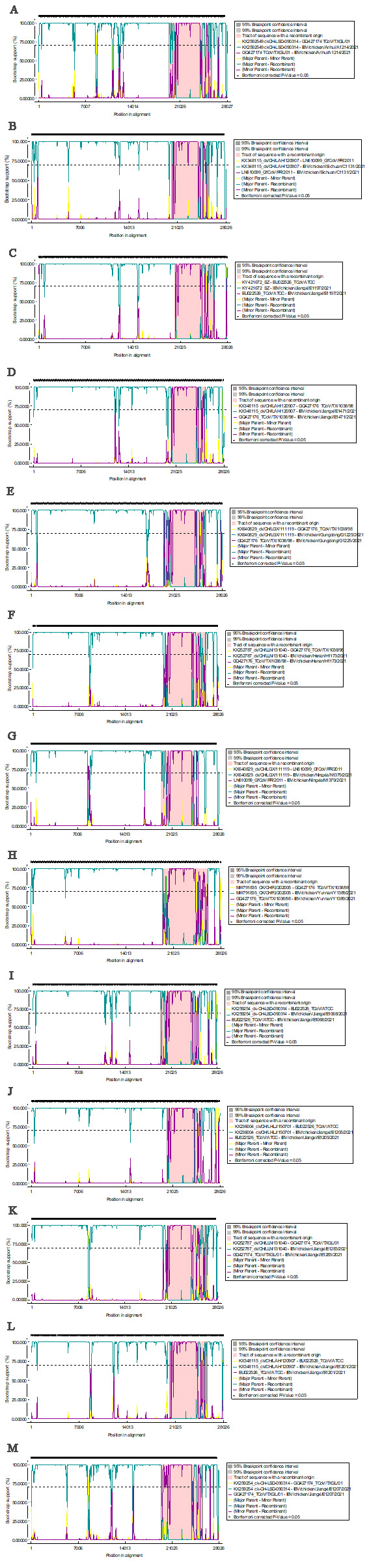

Supplement: Supplementary file 3 [file Image_3.JPEG]

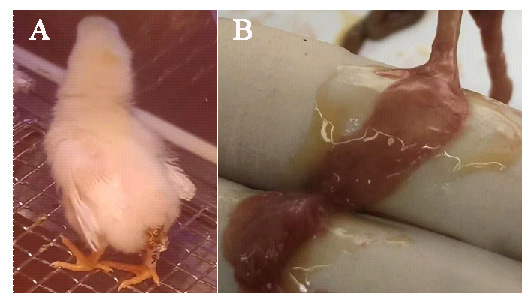

Supplement: Supplementary file 4 [file Image_4.JPEG]
