# Supplementary material for: Characterization of the emerging recombinant infectious bronchitis virus in China
Source: Front Microbiol. 2024 Oct 15;15:1456415. doi: 10.3389/fmicb.2024.1456415 (PMC11518803; doi:10.3389/fmicb.2024.1456415)
Supplement: Supplementary file 5 [file Data_Sheet_1.docx]

**Fig S1 The insertions and deletions of the 13 emerging recombinant IBV strains with reference sequences**

1. IBV/chicken/Jiangxi/E1283/2021 and IBV/chicken/Gungdong/G1225/2021 have deletions of 27 nucleotides and 30 nucleotides in the 1a gene which similar to H120 strain, respectively. (B) Three isolates have deletions of 27 nucleotides in the 3a and 3b genes which similar to ahysx-1strain but different from the common IBV reference strains. (C) The 13 emerging recombinant IBV isolates have two specific deletions of 9 and 3 nucleotides in M gene respectively, similar to ahysx-1 strain but different from the common IBV reference strains. (D) IBV/chicken/Gungdong/G1225/2021 have specific deletion of 3 nucleotides in the *N* gene different from other 12 emerging recombinant IBV isolates, .ahysx-1strain and the common IBV reference strains.

**Fig S2 The M protein transmembrane region prediction of 13 emerging recombinant IBVs**

Three transmembrane regions were predicted at the N-terminal of M protein of 13 isolates.

**Fig S3 The bootscan analysis of the 13 recombinant events**

A - M shows recombination analysis for IBV/chicken/Anhui/A1214/2021, IBV/chicken/Sichuan/C1131/2021, IBV/chicken/Jiangxi/E1197/2021, IBV/chicken/Jiangxi/E1471/2021, IBV/chicken/Gungdong/G1225/2021, IBV/chicken/Henan/H1173/2021, IBV/chicken/Ningxia/N1379/2021, IBV/chicken/Yunnan/Y1389/2021, IBV/chicken/Jiangxi/E1068/2021, IBV/chicken/Jiangxi/E1205/2021, IBV/chicken/Jiangxi/E1283/2021, IBV/chicken/Jiangxi/E1201/2021, IBV/chicken/Jiangxi/E1207/2021 in turn.

**Fig S4 Clinical symptoms of chicks after inoculation**

(A) All chicks appeared paste anus on day 4. (B) At necropsy the intestine revealed congestion.

**Table S1** **Information on primers and probes of RT-qPCR assay for AIV, NDV and ALV-J**

| **Specificity** | **Name of primer/probe** | **Sequence (5’-3’)** | **References** |
| --- | --- | --- | --- |
| AIV | M+25 | AGATGAGTCTTCTAACCGAGGTCG | (Spackman et al., 2002) |
|  | M+64 | FAM-TCAGGCCCCCTCAAAGCCGA-TAMRA |  |
|  | M-124 | TGCAAAAACATCTTCAAGTCTCTG |  |
| NDV | M+4100 | AGTGATGTGCTCGGACCTTC | (Wise et al., 2004) |
|  | M+4169 | FAM-TTCTCTAGCAGTGGGACAGCCTGC-TAMRA |  |
|  | M-4220 | CTGAGGAGAGGCATTTGCTA |  |
| ALV-J | Forward | AGAAAGACCCGGAGAAGAC | Diagnostic techniques for avian leukemia (GB/T 26436-2010) |
|  | *Taq*Man probe | FAM-ATTTTCCGTTTGTCCCAGGGGTGG-TAMRA |  |
|  | Reverse | ACACGTTTCCTGGTTGTT |  |

AIV, avian influenza virus; NDV, Newcastle disease virus; ALV-J, J subgroup avian leukemia virus.

**Table S2 Genome information of 83** **reference sequences from GenBank**

| **Strain** | **Genus** | **Species** | **GenBank accession number** |
| --- | --- | --- | --- |
| Miller M60 | *Alphacoronavirus* | TGEV | DQ811786 |
| 174/06 |  | Canine coronavirus | EU856362 |
| WD1133 |  | Mink coronavirus | HM245926 |
| TN-449 |  | Canine coronavirus | JQ404410 |
| CA08-1/2008 |  | Alpaca respiratory coronavirus | JQ410000 |
| BtNvAlphaCoV/SC2013 |  | Alphacoronavirus | KJ473809 |
| USA/MO/2014/03293 |  | PEDV | KM975741 |
| UF-1/2015 |  | Human coronavirus NL63 | KT381875 |
| UG-FH8 |  | Feline coronavirus | KX722529 |
| TGEV/Mex/145/2008 |  | TGEV | KX900402 |
| Sa-CoV-T14 |  | Alphacoronavirus | KY370053 |
| PC22A |  | PEDV | KY499262 |
| Sm-CoV-X74 |  | Alphacoronavirus | KY967715 |
| FarmA |  | SADs | MF094681 |
| 141388 |  | SADs | MF094687 |
| Mink/China/1/2016 |  | Alphacoronavirus | MF113046 |
| CN0601/14 |  | Human coronavirus NL63 | MG772808 |
| Felix |  | Feline coronavirus | MG893511 |
| WD1127 |  | Mink coronavirus | NC023760 |
| Urbani | *Betacoronavirus* | SARS coronavirus | AY278741 |
| OC43/ATCC VR-759 |  | Human coronavirus | AY585228 |
| HKU1 |  | Human coronavirus | AY597011 |
| A59 |  | Murine hepatitis virus | AY700211 |
| VW572 |  | Porcine hemagglutinating encephalomyelitis virus | DQ011855 |
| HKU3-2 |  | bat SARS coronavirus | DQ084199 |
| MA-15 |  | SARS coronavirus | DQ497008 |
| HKU5-1 |  | Bat coronavirus | EF065509 |
| US/OH1/2003 |  | Sable antelope coronavirus | EF424621 |
| US/OH3/2003 |  | Giraffe coronavirus | EF424623 |
| US/OH3/2006 |  | Calf-giraffe coronavirus | EF424624 |
| P3pp60 |  | SARS coronavirus | FJ882950 |
| 8190 |  | Rat coronavirus | JF792617 |
| EMC/2012 |  | Human betacoronavirus 2c | JX869059 |
| ErinaceusCoV/2012-174/GER/2012 |  | Betacoronavirus Erinaceus/VMC/DEU/2012 | KC545383 |
| Neoromicia/PML-PHE1/RSA/2011 |  | Coronavirus | KC869678 |
| MHV/BHKR lab/USA/icA59 L94P/2012 |  | Murine coronavirus | KF268338 |
| SARS/VeroE6 lab/USA/WTic c1P10/2009 |  | SARS coronavirus wtic-MB | KF514419 |
| Zhejiang2013 |  | Bat Hp-betacoronavirus | KF636752 |
| Jeddah C7149/KSA/2014-04-05 |  | MERS | KM027255 |
| Makkah C9355/KSA/Makkah/2014-04-15 |  | MERS | KM027261 |
| HKU24-R05005I |  | Betacoronavirus | KM349742 |
| ChinaGD01 |  | MERS | KT006149 |
| camel/Riyadh/Ry123/2015 |  | Camel coronavirus HKU23 | KT368891 |
| GCCDC1 356 |  | Rousettus bat coronavirus | KU762338 |
| Jiyuan-84 |  | Bat coronavirus | KY770860 |
| Tokachi09 |  | Equine coronavirus | LC061272 |
| Obihiro12-2 |  | Equine coronavirus | LC061274 |
| W17-18 |  | Water deer coronavirus | MG518518 |
| 4-17-08 |  | Bovine coronavirus | MH043954 |
| HCoV-EMC |  | MERS | MH306207 |
| RaTG13 |  | Bat coronavirus | MN996532 |
| BetaCoV/Wuhan/IPBCAMS-WH-02/2019 |  | Severe acute respiratory syndrome coronavirus 2 | MT019530 |
| SARS-CoV-2/human/PAK/Gilgit1/2020 |  | Severe acute respiratory syndrome coronavirus 2 | MT240479 |
| BCoV-ENT |  | Bovine coronavirus | NC003045 |
| Tor2 |  | SARS coronavirus | NC004718 |
| HKU4-1 |  | Bat coronavirus | NC009019 |
| HKU9-1 |  | Bat coronavirus | NC009021 |
| HKU14 |  | Rabbit coronavirus | NC017083 |
| Wuhan-Hu-1 |  | Severe acute respiratory syndrome coronavirus 2 | NC045512 |
| SARS-CoV-2/human/USA/VA-CAV VAS3N 00014226 01/2022 |  | Severe acute respiratory syndrome coronavirus 2 | OP472825 |
| HKU11-796 | *Deltacoronavirus* | Bulbul coronavirus | FJ376620 |
| HKU20-9243 |  | Wigeon coronavirus | JQ065048 |
| HKU21-8295 |  | Common-moorhen coronavirus | JQ065049 |
| 8734/USA-IA/2014 |  | Porcine deltacoronavirus | KJ567050 |
| PDCoV/USA/Illinois133/2014 |  | Deltacoronavirus | KJ601777 |
| UAE-HKU29 271F |  | Pigeon coronavirus | LC364344 |
| HKU12-600 |  | Thrush coronavirus | NC011549 |
| HKU13-3514 |  | Munia coronavirus | NC011550 |
| HKU19 |  | Night-heron coronavirus | NC016994 |
| TCoV-ATCC | *Gammacoronavirus* | Turkey coronavirus | EU022526 |
| H120 |  | Infectious bronchitis virus | FJ888351 |
| TCoV/TX-GL/01 |  | Turkey coronavirus | GQ427174 |
| HKU22/CF090325 |  | Bottlenose dolphin coronavirus | KF793824 |
| HKU22/CF090331 |  | Bottlenose dolphin coronavirus | KF793826 |
| DK/GD/27/2014 |  | Duck coronavirus | KM454473 |
| GfCoV/FR/2011 |  | Guinea fowl coronavirus | LN610099 |
| ahysx-1 |  | Infectious bronchitis virus | MK142676 |
| Cambridge Bay 2017 |  | Canada goose coronavirus | MK359255 |
| QX |  | Infectious bronchitis virus | MN548289 |
| gammaCoV/Tk/Poland/G160/2016 |  | Turkey coronavirus | MT367412 |
| Beaudette |  | Avian infectious bronchitis virus | NC001451 |
| SW1 |  | Beluga Whale coronavirus | NC010646 |
| Cambridge Bay 2017 |  | Canada goose coronavirus | NC046965 |

**Table S3** **Antigenic epitope prediction analysis o****f S protein of 13 emerging recombinant IBVs**

| **Isolate** | **Cleavage site** | **Epitope number of S protein** | **Average propensity** | **Epitope number of S1 subunit** | **Average propensity** | **Epitope number of S2 subunit** | **Average propensity** |
| --- | --- | --- | --- | --- | --- | --- | --- |
| IBV/chicken/Anhui/A1214/2021 | RTRRS↓ I | 41 | 1.0415 | 19 | 1.0385 | 22 | 1.0440 |
| IBV/chicken/Sichuan/C1131/2021 | RTRRS↓ I | 43 | 1.0413 | 21 | 1.0380 | 22 | 1.0446 |
| IBV/chicken/Jiangxi/E1197/2021 | RTRRS↓ I | 43 | 1.0419 | 21 | 1.0392 | 22 | 1.0437 |
| IBV/chicken/Jiangxi/E1471/2021 | RTRRS↓ I | 42 | 1.0411 | 20 | 1.0370 | 22 | 1.0437 |
| IBV/chicken/Gungdong/G1225/2021 | RTRRS↓ I | 42 | 1.0411 | 20 | 1.0374 | 22 | 1.0438 |
| IBV/chicken/Henan/H1173/2021 | RTRRS↓ I | 44 | 1.0413 | 20 | 1.0378 | 22 | 1.0441 |
| IBV/chicken/Ningxia/N1379/2021 | RTRRS↓ I | 42 | 1.0414 | 19 | 1.0378 | 23 | 1.0439 |
| IBV/chicken/Yunnan/Y1389/2021 | RTRRS↓ I | 42 | 1.0420 | 20 | 1.0389 | 22 | 1.0446 |
| IBV/chicken/Jiangxi/E1068/2021 | RTRRS↓ I | 42 | 1.0413 | 20 | 1.0379 | 22 | 1.0437 |
| IBV/chicken/Jiangxi/E1205/2021 | RTRRS↓ I | 43 | 1.0419 | 21 | 1.0392 | 22 | 1.0437 |
| IBV/chicken/Jiangxi/E1283/2021 | RTRRS↓ I | 41 | 1.0416 | 19 | 1.0381 | 22 | 1.0441 |
| IBV/chicken/Jiangxi/E1201/2021 | RTRRS↓ I | 42 | 1.0413 | 20 | 1.0379 | 22 | 1.0437 |
| IBV/chicken/Jiangxi/E1207/2021 | RTRRS↓ I | 43 | 1.0419 | 21 | 1.0392 | 22 | 1.0437 |

**Table S4 The M protein transmembrane region prediction of 13 emerging recombinant IBVs**

| **Strain** | **Length (amino acid )** | **ExpAA** | **First 60** | **Predicted Hel** | **Topology** |
| --- | --- | --- | --- | --- | --- |
| ahysx-1 | 222 | 64.02 | 35.71 | 3 | o15-37i44-66o76-98i |
| IBV/chicken/Anhui/A1214/2021 | 223 | 63.82 | 34.41 | 3 | o20-39i51-70o74-96i |
| IBV/chicken/Sichuan/C1131/2021 | 222 | 64.24 | 36.28 | 3 | o15-37i44-66o76-98i |
| IBV/chicken/Jiangxi/E1068/2021 | 222 | 64.02 | 35.71 | 3 | o15-37i44-66o76-98i |
| IBV/chicken/Jiangxi/E1197/2021 | 222 | 64.02 | 35.71 | 3 | o15-37i44-66o76-98i |
| IBV/chicken/Jiangxi/E1201/2021 | 222 | 64.02 | 35.71 | 3 | o15-37i44-66o76-98i |
| IBV/chicken/Jiangxi/E1205/2021 | 222 | 64.02 | 35.71 | 3 | o15-37i44-66o76-98i |
| IBV/chicken/Jiangxi/E1207/2021 | 222 | 64.02 | 35.71 | 3 | o15-37i44-66o76-98i |
| IBV/chicken/Jiangxi/E1283/2021 | 222 | 64.02 | 35.71 | 3 | o15-37i44-66o76-98i |
| IBV/chicken/Jiangxi/E1471/2021 | 222 | 64.02 | 35.71 | 3 | o15-37i44-66o76-98i |
| IBV/chicken/Henan/H1173/2021 | 222 | 64.02 | 35.71 | 3 | o15-37i44-66o76-98i |
| IBV/chicken/Guangdong/G1225/2021 | 222 | 64.14 | 35.85 | 3 | o15-37i44-66o76-98i |
| IBV/chicken/Ningxia/N1379/2021 | 223 | 63.84 | 34.29 | 3 | o20-39i51-70o74-96i |
| IBV/chicken/Yunnan/Y1398/2021 | 222 | 64.02 | 35.71 | 3 | o15-37i44-66o76-98i |
